# Effects on cardiorespiratory fitness of moderate-intensity training vs. energy-matched training with increasing intensity

**DOI:** 10.3389/fspor.2023.1298877

**Published:** 2024-01-04

**Authors:** Marcel Reuter, Friederike Rosenberger, Andreas Barz, Andreas Venhorst, Laura Blanz, Kai Roecker, Tim Meyer

**Affiliations:** ^1^ Institute of Sports and Preventive Medicine, University of Saarland, Saarbrücken, Germany; ^2^ Department of Applied Training Science, German University of Applied Sciences for Prevention and Health Management (DHfPG), Saarbrücken, Germany; ^3^ Department of Medical Oncology, National Center for Tumor Diseases (NCT), Heidelberg, Germany; ^4^ Institute for Health Promotion and Exercise Medicine (IfAG), Furtwangen University, Furtwangen, Germany

**Keywords:** aerobic fitness, VO_2max_, HIIT, energy expenditure, endurance training, running economy

## Abstract

**Introduction:**

The present study investigated the role of training intensity in the dose–response relationship between endurance training and cardiorespiratory fitness (CRF). The hypothesis was that beginners would benefit from an increase in training intensity after an initial training phase, even if the energy expenditure was not altered. For this purpose, 26 weeks of continuous moderate training (control group, CON) was compared to training with gradually increasing intensity (intervention group, INC) but constant energy expenditure.

**Methods:**

Thirty-one healthy, untrained subjects (13 men, 18 women; 46 ± 8 years; body mass index 25.4 ± 3.3 kg m^−2^; maximum oxygen uptake, VO_2max_ 34 ± 4 ml min^−1^ kg^−1^) trained for 10 weeks with moderate intensity [3 days/week for 50 min/session at 55% heart rate reserve (HR_reserve_)] before allocation to one of two groups. A minimization technique was used to ensure homogeneous groups. While group CON continued with moderate intensity for 16 weeks, the INC group trained at 70% HR_reserve_ for 8 weeks and thereafter participated in a 4 × 4 training program (high-intensity interval training, HIIT) for 8 weeks. Constant energy expenditure was ensured by indirect calorimetry and corresponding adjustment of the training volume. Treadmill tests were performed at baseline and after 10, 18, and 26 weeks.

**Results:**

The INC group showed improved VO_2max_ (3.4 ± 2.7 ml kg^−1^ min^−1^) to a significantly greater degree than the CON group (0.4 ± 2.9 ml kg^−1^ min^−1^) (*P* = 0.020). In addition, the INC group exhibited improved V_max_ (1.7 ± 0.7 km h^−1^) to a significantly greater degree than the CON group (1.0 ± 0.5 km h^−1^) (*P* = 0.001). The reduction of resting HR was significantly larger in the INC group (7 ± 4 bpm) than in the CON group (2 ± 6 bpm) (*P* = 0.001). The mean heart rate in the submaximal exercise test was reduced significantly in the CON group (5 ± 6 bpm; *P* = 0.007) and in the INC group (8 ± 7 bpm; *P* = 0.001), without a significant interaction between group and time point.

**Conclusion:**

Increasing intensity leads to greater adaptations in CRF than continuing with moderate intensity, even without increased energy expenditure. After 26 weeks of training in the moderate- and higher-intensity domain, energy-matched HIIT elicited further adaptations in cardiorespiratory fitness. Thus, training intensity plays a crucial role in the dose–response relationship between endurance training and fitness in untrained but healthy individuals.

**Clinical Trial Registration:**

https://www.drks.de/DRKS00031445, identifier DRKS00031445.

## Introduction

Regular physical activity leads to higher levels of fitness and lower risk for cardiovascular diseases (CVDs) (1, 2). Physical fitness is an important and independent prognostic factor that can be divided into physiological (e.g., maximum oxygen uptake, VO_2max_) and functional (e.g., maximum velocity, V_max_) components (3). Beginners to training without pre-existing conditions are often referred to the World Health Organization (WHO) recommendations for physical activity as guidelines for their training. For adults aged 18–64 years, the WHO recommends at least 150 min of moderate-intensity or at least 75 min of vigorous-intensity aerobic physical activity per week, or an equivalent combination of moderate- and vigorous-intensity activity throughout the week (4).

The extent to which variations in either volume or intensity lead to different outcomes in terms of cardiorespiratory fitness (CRF) has been the subject of numerous studies (5–7). Training goals and individual levels of fitness guide decisions about specific training methods to be used. Especially in beginners, training with moderate intensity seems to be adequate to improve fitness (3), which is frequently represented by VO_2max_ (8).

Training load can be quantified by calculating the energy expenditure (EE), which is often expressed as the caloric expenditure (kcal). However, without any alterations to the training load, further adaptions become less likely over time (9). These findings raise the question of which training load modification is most effective. One common strategy is to keep the intensity constant but increase the overall volume (10) through longer sessions or a higher number of sessions (11). Another approach is to increase training intensity. In this context, high-intensity interval training (HIIT) is frequently seen as an effective training method (7, 12–14). A common HIIT training protocol is the “4 × 4” method. This involves four intervals, each lasting 4 min, at a heart rate of 95% HR_max_. The recovery time between each interval is 3 min at a heart rate of 70% HR_max_ (15).

The differences between moderate-intensity continuous training (CON) and HIIT were examined in a meta-analysis by Poon et al. (16). They found that interval training has a significantly greater effect on improvements in VO_2max_ than training with moderate continuous intensity in middle-aged adults.

There is evidence that higher training intensities lead to higher VO_2max_ than isocaloric moderate-intensity training in obese adults (17).

One methodological challenge when comparing CON with HIIT is that the total energy expenditure under each intervention is not always controlled for (16, 18, 19). In such studies, it remains unclear whether the effects can be attributed to higher training intensity or to a higher total energy expenditure. Additionally, studies rarely last longer than 12 weeks (5, 20). Finally, they often neglect the effects on submaximal parameters despite their relevance to CRF (21). To our knowledge, no study has investigated the effects of a gradual increase in training intensity with constant energy expenditure on maximal and submaximal parameters in healthy inactive adults. We therefore analyzed the differences between 6 months of continuous moderate training (control group, CON) and training with a gradual training intensity increase but constant training volume (intervention group, INC) in terms of their effect on CRF. We hypothesized that INC would lead to more pronounced effects on CRF over time than CON.

Other researchers have shown that training with higher intensities does not lead to different degrees of adaption of lactate threshold or running economy (RE) (7, 22). Therefore, we hypothesized that the effects on submaximal parameters would be identical for both groups.

## Materials and methods

### Study design

**Figure 1:**
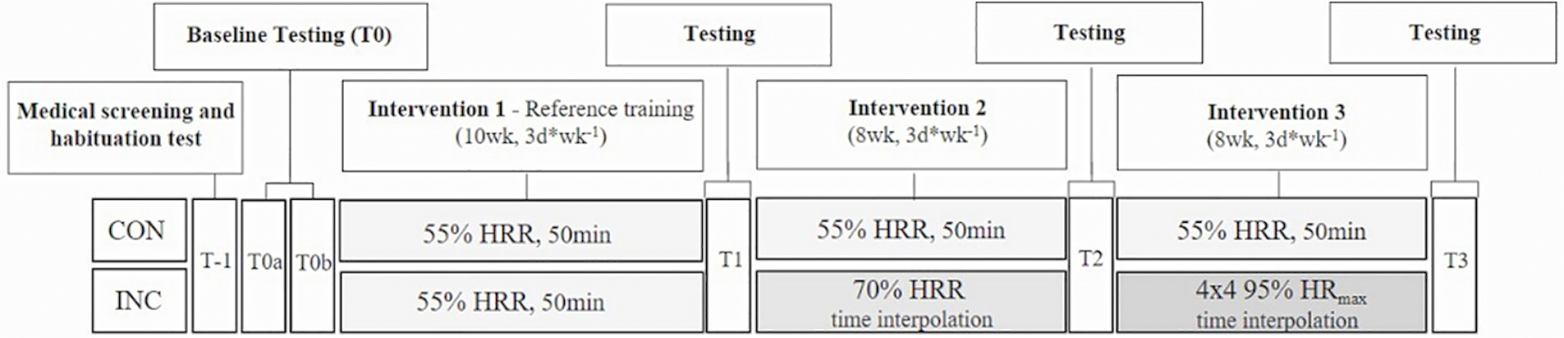
Study design.

Our study was a two-arm randomized training intervention trial (Figure 1). Participants in one arm continuously performed moderate-intensity endurance training over 26 weeks (CON), while those in the other arm increased their training intensity from moderate to vigorous after 10 weeks and to high-intensity interval training after 18 weeks (INC). EE was matched within subjects throughout all stages.

Outcomes were assessed at baseline (T0) and after 10 (T1), 18 (T2), and 26 (T3) weeks of training. All participants gave written informed consent prior to participation. The study procedures were in accordance with the Declaration of Helsinki, and the study was approved by the Ethics Committee of the Medical Association of Saarland (identification number 219/19). This data set was also used in a different research approach to analyze the training effects at the individual level by comparing the response rate between groups (23).

### Participants

**Figure 2:**
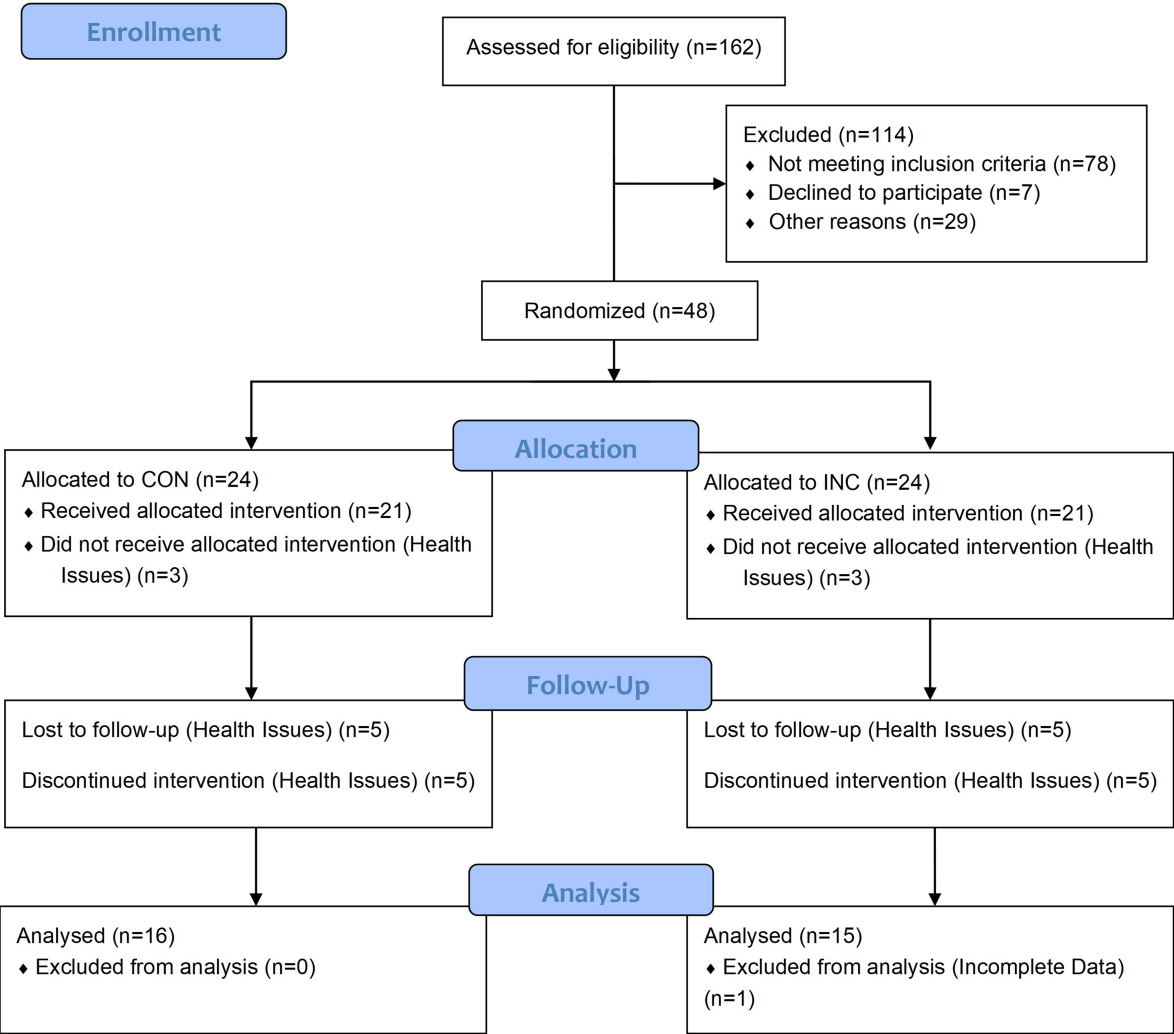
Flow diagram.

Participants were recruited through a newspaper advertisement (Figure 2). The inclusion criteria were: age 30–60 years, untrained (last 6 months: <1 h/week endurance-type physical activity), and non-smokers. The exclusion criteria were: body mass index (BMI) > 30 kg m^−2^, resting blood pressure (BP_rest_) ≥ 160/100 mmHg), total cholesterol ≥ 300 mg dl^−1^, VO_2max_ > 50 ml kg^−1^ min^−1^ for men or >45 ml kg^−1^ min^−1^ for women, iron deficiency (ferritin ≤ 34 ng ml^−1^), thyroid dysfunction (TSH ≤ 0.34 mU L^−1^; ≥4.0 ng ml^−1^), medications with potential influence on target parameters (e.g., beta-blockers), and pregnancy. Participants’ characteristics are given in Table 1. There were no between-group differences at baseline for any of these variables (*P* > 0.05).

**Table 1:** Main participant characteristics and outcomes.

	T0		T1		T2		T3		ANOVA	
	CON	INC	CON	INC	CON	INC	CON	INC	Time × group	Time
Sex										
Men	6 (37%)	7 (47%)								
Women	10 (63%)	8 (53%)								
Anthropometric data										
Age (years)	46.9 ± 7.8	45.9 ± 9.0								
Height (cm)	170.6 ± 9.9	171.2 ± 5.3								
Weight (kg)	75 ± 16	75 ± 12	74 ± 15	74 ± 11	74 ± 15	73 ± 10	74 ± 16	71 ± 10	NS	*P* = 0.001
BMI (kg m^−2^)	25.5 ± 3.3	25.4 ± 3.5	25.2 ± 3.2	25.3 ± 3.3	25.2 ± 3.2	24.8 ± 3.1	25.0 ± 3.4	24.3 ± 3.0	NS	NS
Body fat (%)	23 ± 4	22 ± 4	23 ± 4	22 ± 4	23 ± 5	21 ± 4	22 ± 5	20 ± 4	NS	*P* = 0.001
Hemodynamic characteristics at rest										
HR (bpm)	69 ± 9	68 ± 6	65 ± 8	67 ± 6	67 ± 8	64 ± 5	67 ± 8	61 ± 5	*P* = 0.001	*P* = 0.000
Systolic BP (mmHg)	119 ± 7	121 ± 10	118 ± 8	119 ± 8	118 ± 12	117 ± 8	122 ± 11	118 ± 9	NS	NS
Diastolic BP (mmHg)	79 ± 7	81 ± 8	78 ± 5	79 ± 6	79 ± 9	80 ± 6	81 ± 7	79 ± 7	NS	NS
Peak exercise performance										
VO_2max_ (ml min^−1^)	2,596 ± 545	2,524 ± 514	2,625 ± 576	2,610 ± 545	2,514 ± 530	2,544 ± 513	2,584 ± 577	2,666 ± 536	NS	*P* = 0.003
VO_2max_ (ml min^−1^ kg^−1^)	34.9 ± 3.6	34.0 ± 5.2	35.6 ± 4.5	35.2 ± 5.5	34.1 ± 3.9	35.0 ± 5.1	35.3 ± 4.0	37.3 ± 4.9	*P* = 0.02	*P* = 0.001
V_max_ (km h^−1^)	11.3 ± 1.4	10.9 ± 1.4	12.1 ± 1.4	11.7 ± 1.5	12.3 ± 1.6	12.1 ± 1.5	12.3 ± 1.6	12.6 ± 1.4	*P* = 0.000	*P* = 0.000
HR_max_ (bpm)	186 ± 12	182 ± 13	184 ± 12	184 ± 13	186 ± 12	180 ± 12	185 ± 11	181 ± 12	NS	NS
La_max_ (mmol L^−1^)	9.5 ± 2.2	8.4 ± 2.1	9.8 ± 2.1	9.4 ± 3.0	9.8 ± 1.8	8.9 ± 2.8	9.5 ± 2.2	9.4 ± 2.0	NS	NS
RER_max_	1.2 ± 0.1	1.2 ± 0.1	1.2 ± 0.1	1.2 ± 0.0	1.3 ± 0.0	1.2 ± 0.1	1.3 ± 0.1	1.3 ± 0.1	NS	NS
Submaximal exercise performance										
RE (ml kg^−1^ km^−1^)	161 ± 50	157 ± 75	157 ± 52	148 ± 74	148 ± 46	150 ± 67	150 ± 47	152 ± 73	NS	*P* = 0.004

T0, T0a and T0b; HR, heart rate; BP, blood pressure; La_max_, maximum lactate; RER_max_, maximum respiratory exchange ratio; NS, not significant.

Values are mean ± SD.

### Testing procedures

Three baseline exercise tests were performed for habituation (T1) and to assess the day-to-day variability of VO_2max_ (T0a, T0b). At T1, all participants underwent a medical examination, blood sampling, resting and exercise electrocardiogram (ECG), and cardiorespiratory exercise testing on a treadmill (type ELG 70, Woodway GmbH, Weil am Rhein, Germany). T1 was not included in the data, and values for T0 represent the mean of values for T0a and T0b. Before exercise testing, height and body weight were measured. Body fat percentage was assessed by a 10-site skinfold method with a Harpenden caliper. Resting heart rate (HR_rest_) and BP_rest_ were assessed in a supine position after a 10-min resting period at the left and right arms.

### Randomization

At T1, subjects were allocated to either CON or INC using a minimization technique (24). Factors for balancing were age, sex, baseline level of and improvement in VO_2max_ (ml min^−1^), and VO_2max_ response (yes/no, double-weighted). A response was defined as an increase in VO_2max_ (ml min^−1^) at T1 that exceeded the within-subject variability (iV) (25). iV represents the relative day-to-day variability of VO_2max_ between T0a and T0b and was calculated as follows:

iV=|T0a−T0b|/T0

**Maximal exercise tests** were performed on a treadmill with a constant inclination of 0.5%. The test protocol was a combination of a graded exercise test (GXT) and a ramp protocol to allow for the measurement of submaximal parameters (HR, lactate), RE, V_max_, and VO_2max_ (26). All tests started at 4.0 km h^−1^. Speed was increased incrementally by 1.0 km h^−1^ every 3 min, with approximately 30 s of rest after each stage for lactate sampling. The speed at which participants transitioned from walking to running and from the GXT to the ramp protocol was standardized within each subject over all tests. The last 3-min stage was completed when the respiratory exchange ratio (RER) exceeded 0.95 for at least 30 s, as it was assumed that this approximates lactate values of baseline lactate + 1 mmol. Subsequently, participants switched to a ramp protocol with a speed increment of 0.8 km h^−1^ per minute until voluntary exhaustion. V_max_ is the maximal running velocity at the point of test termination.

**Gas exchange measurements** were performed continuously using a breath-by-breath system (MetaLyzer 3B, Cortex Biophysik GmbH, Leipzig, Germany). Only tests that met at least two of the following criteria were included: (a) HR_max_ ≥ (220-age), (b) maximal blood lactate concentration >8 mmol L^−1^, and (c) maximal RER >1.1 (27).

**Lactate samples** were taken at rest, during each break between GXT stages, and during the post-exercise period (first, third, and fifth minute) from the hyperemic earlobe. An enzymatic–amperometric method was used to analyze the samples (Super GL, Rolf Greiner Biochemica, Flacht, Germany). To analyze changes in submaximal heart rate and lactate during the GXT, measurements for 4, 5, 6, and 7 km h^−1^ were compared, as all participants completed these stages. V̇O_2_ at the individual penultimate stage was taken to calculate RE as participants’ RER was <0.95 at this stage and the maximal lactate steady state was very likely not surpassed. RE was calculated as the oxygen cost per kilometer of running relative to bodyweight (ml kg^−1^ km^−1^) (28).

### Exercise training intervention

The training program consisted of 26 weeks of walking or jogging, 3 days/week. Training until T1 was identical for the CON and INC groups: 3 days/week for 50 min/session at 55% heart rate reserve (HR_reserve_, %HRR). The CON group continued their training. The INC group increased intensity to 70% HRR between T1 and T2. At T2, the INC group changed to a 4 × 4 interval training program, consisting of a 10-min warm-up at 70% HR_max_, 4 × 4-min intervals at 95% HR_max_ with 3 × 3-min “breaks” in between at 70% HR_max_, and a cool-down at 70% HR_max_ (7). Within subjects, EE was standardized over all intervention phases by adjusting the training time per session after T1 (mean duration, 42 min/session) and the length of the cool-down after T2 (mean duration, 35 min/session).

For the approximate calculation of EE, respective oxygen uptake at the individual exercise heart rate (55% HRR, 70% HRR, 70% HR_max_, 95% HR_max_) was measured during the treadmill test. Total EE per training session (TS) was calculated by use of an average caloric equivalent (4.85 kcal L^−1^ O_2_) (29) and the training time per session.

Participants were given a heart rate monitor with a chest strap (Sigma R1 Duo + ID.Free, Sigma-Elektro GmbH, Neustadt) to monitor their training. Using data from these monitors, the mean training heart rate and the mean heart rate for the high-intensity intervals (for HIIT) were evaluated and compared to the prescribed training heart rates to evaluate adherence.

### Statistical analyses

G*Power (version 3.1.9.4) was used to calculate the sample size per group for an ANOVA [main and interaction effects on VO_2max_ (ml min^−1^ kg^−1^)] with *α* = 0.05, 80% power, two groups, and two test time points. For this purpose, we used an effect size from a comparison between groups from two out of four arms of a previous INC training intervention study by Helgerud et al. (7): dppc2 = 0.547. The required sample was *n* = 29 in total, or 15 subjects per group. Taking into account an estimated dropout rate of 28% (9), the aim was to include 20 participants per group.

The Statistical Package for the Social Sciences (SPSS v27, IBM, USA) was used for statistical analysis. Test assumptions were met. Group differences at baseline were examined using *t*-tests for independent samples. Mixed ANOVAs were used to analyze between- and within-subject effects of the true value of the change in performance. The Greenhouse–Geisser epsilon adjustment was made when sphericity was violated. Partial eta-squared (*η*_p_^2^) was used as a measure of effect size. The limits for effect sizes were set at 0.01 (small effect), 0.06 (medium effect), and 0.14 (large effect) (30). Mixed ANOVAs with the three factors of group, time, and velocity were used to analyze changes in heart rate and blood lactate concentration during the GXT protocol. For all other parameters, mixed ANOVAs with the factors of group and time were conducted. A *P*-value of <0.05 was considered statistically significant. Data are expressed as the mean ± standard deviation (SD).

For RE, there were two outliers in the data, as assessed by inspection of a boxplot for values greater than 1.5 box lengths from the edge of the box. Both these outliers (*n* = 1) were removed from the analysis.

## Results

### Compliance

The CON group completed 79.6 ± 6.95 training sessions and the INC group 79.8 ± 8.09. There were no statistically significant between-group differences in training frequency, adherence to exercise HR, or adherence to EE (*P* > 0.05). The average EE per training session estimated by indirect calorimetry was 401 ± 105 kcal (CON: 403 ± 105; INC: 398 ± 104; *P* = 0.914). For the CON group, adherence to training EE was 98 ± 6% up to T2 and 99 ± 5% up to T3. For the INC group, it was 101 ± 5% up to T2 and 102 ± 8% up to T3. Average exercise HR up to T1 was 100 ± 2% (CON) and 101 ± 2% (INC) of prescribed HR. In T2, it was 101 ± 4% (CON) and 99 ± 1% (INC). In T3, adherence to exercise HR for CON was 99 ± 1% and adherence to the exercise HR of 95% HR_max_ for INC was 97 ± 2%.

### Training adaptions

#### VO_2max_

**Figure 3:**
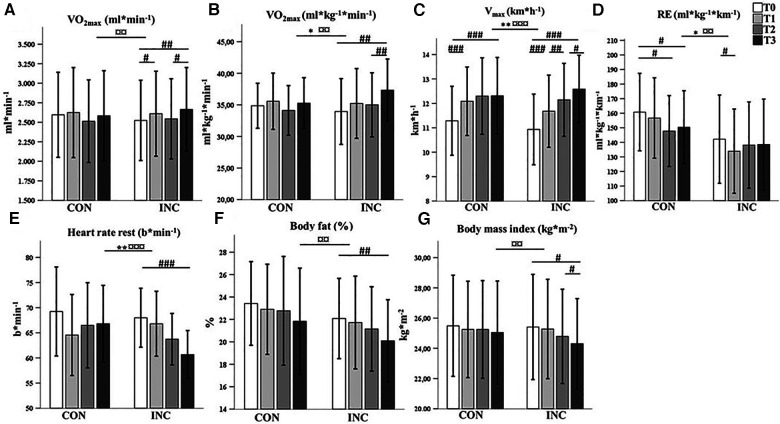
Comparison of mean (SD) (**A**) VO_2max_ (ml min^−1^), (**B**) VO_2max_ (ml min^−1^ kg^−1^), (**C**) V_max_ (km h^−1^), (**D**) RE (ml kg^−1^ km^−1^), (**E**) resting heart rate (bpm), (**F**) body fat (%), and (**G**) BMI (kg m^−2^) between CON and INC and between time points (T0, T1, T2, and T3). *P < 0.05, **P < 0.01, and ***P < 0.001 for group × time interaction. ^¤^*P* < 0.05, ^¤¤^*P* < 0.01, ^¤¤¤^*P* < 0.001 for the main effect of time. ^#^*P* < 0.05, ^##^*P* < 0.01, ^###^*P* < 0.001 for the simple effect of time for the groups.

Changes in VO_2max_ are presented in Figure 3. With regard to relative VO_2max_ (ml min^−1^ kg^−1^), there was a significant interaction of time and intervention (*F*_2.1,61.3_ = 4.1, *P* = 0.020, *η*_p_^2^* = *0.12)*.* Specifically, there was a significant increase over time for INC (*F*_1.9, 27.1_ = 9.4, *P* = 0.001, *η*_p_^2^* *= 0.40) but not for CON (*F*_2.1, 30.8_ = 1.9, *P* = 0.170, *η*_p_^2^* *= 0.11). The improvement between T2 and T3 (HIIT) was significant for INC (*P* = 0.002).

For absolute VO_2max_ (ml min^−1^), the effect of the interaction between time and intervention did not reach statistical significance (*F*_2,3, 67_* = *3.0, *P* = 0.051, *η*_p_^2^* = *0.09), although this value increased over time for INC (*F*_3,42_* = *6.5*, P* = 0.001, *η*_p_^2^* = *0.32).

#### V_max_

There was a statistically significant interaction of time and intervention (*F*_2.2,65.2_ = 7.2, *P* = 0.001, *η*_p_^2^ = 0.2). The changes in V_max_ were significant for CON (*F_3,45_ = *52.8*, P = *0.000, *η_p_*^2^* = *0.78) and INC (*F*_3,42_* = *54.0, *P = *0.000, *η_p_*^2^* = *0.79). Table 1 provides an overview of peak exercise performance parameters.

### Submaximal exercise test

**Figure 4:**
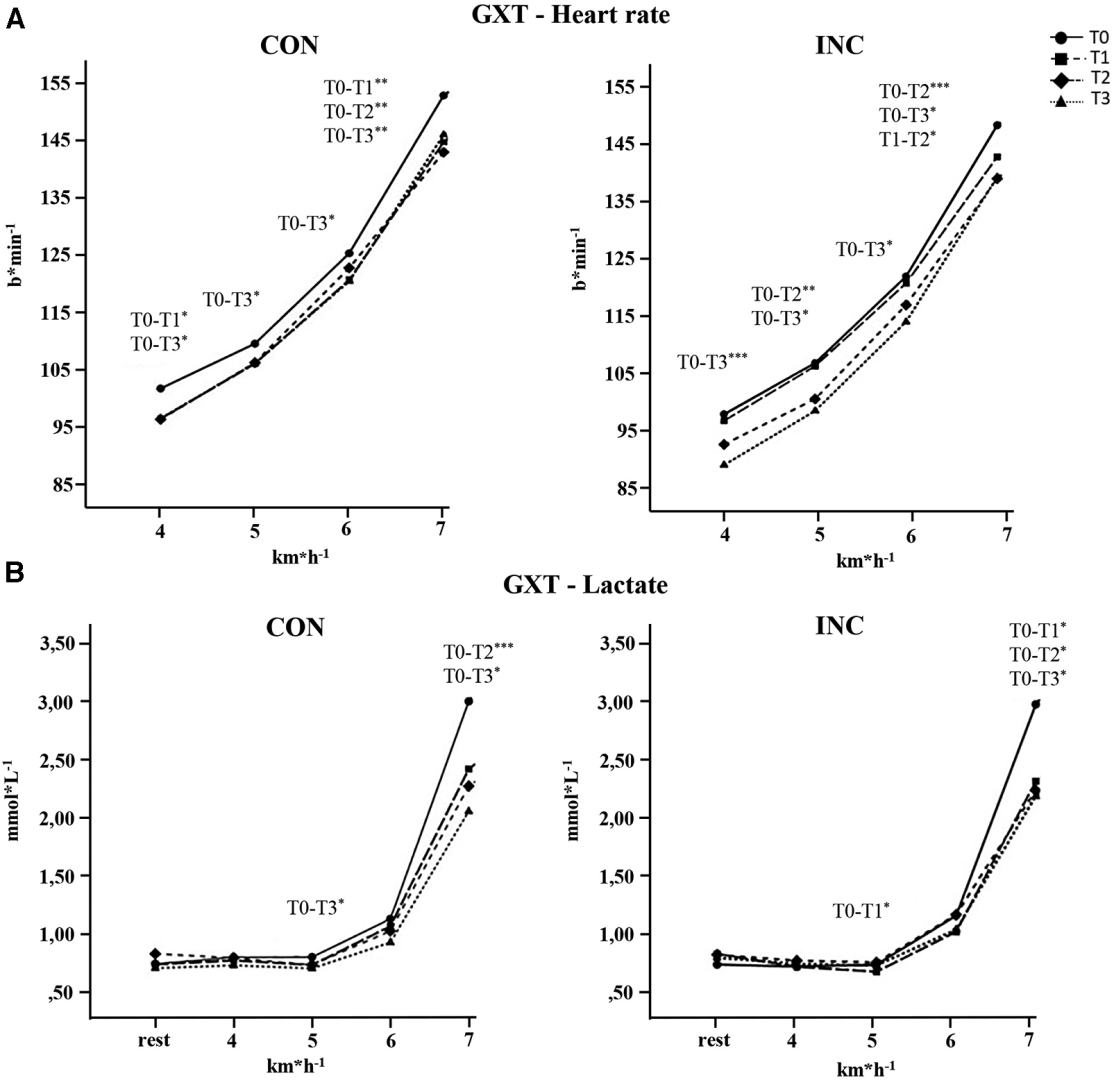
Means and SDs during GXT for (**A**) heart rate and (**B**) lactate for the CON and INC groups. *P < 0.05, **P < .01, ***P < 0.001.

Changes in HR and lactate are presented in Figure 4. For exercise HR, there was no statistically significant three-way interaction between group, time, and running velocity (*F*_5.1, 148.6_ = 0.45, *P* = 0.908, *η*_p_^2^ = 0.02). The mean GXT heart rate was reduced significantly in CON by 5 ± 6 bpm (*P* = 0.007, *η*_p_^2^* = *0.23) and in INC by 8 ± 7 bpm (*P* = 0.001, *η*_p_^2^* *= 0.37).

The lactate value for 7 km h^−1^ was reduced in CON by 1 ± 0.5 mmol L^−1^ and in INC by 0.8 ± 0.9 mmol L^−1^.

There was a statistically significant interaction between intervention and time in their effects on RE (*F*_2.3, 64.5_ = 3.185, *P* = 0.041, *η*_p_^2^* *= 0.1).

### Body composition and resting HR

Changes in body composition are shown in Figure 3. There was no significant interaction of group and time in their effect on bodyweight (*F*_1.7, 49.4_ = 2.3, *P* =0 .116, *η*_p_^2^ = 0.07) or body fat (*F*_2.2, 60.6 _= 0.2, *P* = 0.879, *η*_p_^2^ = 0.01).

Changes in resting HR are shown in Figure 3. The reduction in resting HR was higher in INC than in CON (*F*_3, 87 _= 6.3, *P* = 0.001, *η*_p_^2^ = 0.18).

## Discussion

The main aim of this study was to determine the effect on CRF of increasing intensity while maintaining EE.

### Cardiorespiratory fitness

Our main finding was that 26 weeks of training with an average EE of 400.5 ± 104.5 kcal per training session led to more pronounced effects on CRF over time in the INC group than in the CON group. Increasing the intensity has proven to be more effective in increasing V_max_ and VO_2max_. In accordance with our hypothesis, there were no significant group differences in the reduction of HR or lactate values during the GXT. Our findings show that an increase in training intensity can lead to larger effects even when the energy expenditure per session remains constant.

The overall improvement in V_max_ was 1.7 km h^−1^ in the INC group and 1.0 km h^−1^ in the CON group. Although both groups significantly increased their maximal running velocity, training with increased intensity was found to be more effective. Training with moderate intensity elicited meaningful improvements only in the first 10 weeks of training (0.8 km h^−1^). For the INC group, each increase in intensity led to further significant improvements in V_max_.

In our study, VO_2max_ (ml min^−1^) increased between T0 and T1 (CON: 1.1%; INC: 3.4%), decreased between T1 and T2 (CON: −4.2%; INC: −2.5%), and increased again between T2 and T3 (CON: 2.8%; INC: 4.8%). Parameters of maximal exhaustion were constant over all tests and can therefore be ruled out as a contributing factor to this observation. Comparable studies have found improvements of approximately 190 ml min^−1^ after 12 weeks of moderate training (9). In this study, the average increase after the same time was 57 ml min^−1^.

One explanation for this relatively small adaption, as well as the reduction in VO_2max_ between T1 and T2, is a potential reduction of habitual physical activity that might have occurred in both groups. This could have influenced the response in VO_2max_ negatively (31) but was less pronounced in the INC group than in the CON group, which can be explained by the overall greater training stimulus induced by higher intensity.

It is conceivable that the intervention, especially between T1 and T2, represented an insufficient increase in physical activity compared to baseline. This might also explain why only HIIT has been shown to be effective in improving VO_2max_, as it represented a greater stimulus on the aerobic system, and near-maximal efforts in particular have been shown to be able to elicit adaption of aerobic capacity (32). With regard to VO_2max_ (ml min^−1^ kg^−1^), training at higher intensities led to an overall larger improvement than continuous moderate-intensity training (CON: 1.2%; INC: 10.0%). Our study has shown that improvements in V_max_ can be achieved without an increase of VO_2max_. This observation has also been made in previous studies (5) and can be explained by improvement in submaximal running efficiency (33, 34).

We measured RE and found a larger improvement in the CON group. This can be explained by the fact that the subjects in the CON arm spend more time running with slower velocities in training and could therefore develop more efficient movement patterns for submaximal velocities.

### Submaximal parameters

In terms of other submaximal parameters describing CRF, we found no difference in adaption between groups. Lactate values and submaximal heart rate during the GXT decreased for both groups. Analysis of the individual velocities suggests a downward shift in the heart rate curve and a rightward shift in the lactate curve, which are indicators of cardiac as well as metabolic adaptation (21, 35).

In our study, training intensities were derived from heart rate, as this is the most commonly available measure in practical settings. Despite its high practicality, this approach can lead to considerable between-subject variation in metabolic strain even for similar prescriptions of training heart rate (36). This is why some authors suggest prescribing intensities as percentages of physiologically metabolic thresholds as opposed to maximum heart rate to elicit more homogeneous responses of CRF (37).

### Body mass

Both moderate endurance training and training with a progressive increase in intensity induced some weight reduction. In this study, weight was significantly reduced over time in both groups. This is congruent with the data presented in a meta-analysis by Wewege et al. (19). However, there was also a significant reduction in BMI with HIIT (from T2 to T3), which can be explained by excessive post-exercise oxygen consumption (38). This indicates that increasing exercise intensity without higher EE does not provide additional benefits for weight loss if the intensity does not reach the high-intensity domain.

### Heart rate

Training with increased intensity (INC) has proven to be more effective in reducing HR_rest_ but not submaximal HR. This observation leads to the conclusion that the training completed by the INC group elicited an increase in vagal tone that diminishes as soon as the heart rate is elevated (35). We interpret this as an adaption primarily of the autonomic rather than the cardiac system in the INC group. There was no further decrease in HR_rest_ for the CON group after T1, whereas there was a progressive decrease with increasing intensity for the INC group.

### Limitations

Due to the long duration of this study, as well as the strict exclusion criteria, the dropout rate was high (35%). Despite the high dropout rate, we reached the intended sample size calculated. The number of dropouts was similar in both groups and primarily related to injuries or other health reasons unrelated to the study interventions.

The study was well controlled using adaptive randomization, and the intervention duration of 26 weeks was longer than those of comparable studies (19). However, biological factors and technical errors may have led to considerable day-to-day variability in VO_2max_. In the literature, this variability is reported to be 5.6% (6, 39). In our study, the day-to-day variability was determined individually by repeating measurements between T0a and T0b. This variability averaged 4.2 ± 2.7%. The treadmill tests were conducted in accordance with the ACSM guidelines (27).

Although subjects were advised not to change dietary habits or other physical activity over the course of the intervention, a potential influence of lifestyle changes cannot be entirely ruled out.

## Conclusion

Our study has shown that the choice of training intensity seems to play a crucial role in the dose–response relationship between training and fitness. We were able to show that 26 weeks of endurance training led to several improvements in performance and fitness indicators (V_max_, relative VO_2max_, HR_rest_, and BMI). By progressively increasing the intensity after a further 8 weeks with 70% HRR and 8 weeks of HIIT with 95% HR_max_, positive adaptations continued. In this context, we found evidence that HIIT is likely to be an effective way for untrained healthy runners to improve CRF after an initial phase of training with moderate-to-vigorous intensity. To continuously achieve further adequate training adaptions, the training intensity should be increased over time. Our data also show that improvements in V_max_ without increases in VO_2max_ must be considered normal.

## Data Availability

The raw data supporting the conclusions of this article will be made available by the authors without undue reservation.
